# Preventive effects of low‐intensity endurance exercise for severe hyperglycemia‐induced capillary regression in non‐obese type 2 diabetes rat skeletal muscle

**DOI:** 10.14814/phy2.14712

**Published:** 2021-01-19

**Authors:** Takeshi Morifuji, Minoru Tanaka, Ryosuke Nakanishi, Takumi Hirabayashi, Hiroyo Kondo, Hidemi Fujino

**Affiliations:** ^1^ Department of Rehabilitation Science Kobe University Graduate School of Health Sciences Kobe Japan; ^2^ Department of Physical Therapy Josai International University Tougane Japan; ^3^ Department of Rehabilitation Science Osaka Health Science University Osaka Japan; ^4^ Department of Physical Therapy Faculty of Rehabilitation Kobe international University Kobe Japan; ^5^ Department of Food Science and Nutrition Nagoya Women’s University Nagoya Japan

**Keywords:** capillary regression, low‐intensity exercise, severe hyperglycemia, type 2 diabetes

## Abstract

Although endurance exercise is effective for reducing diabetes‐related capillary regression, it is difficult to prescribe high‐intensity endurance exercise due to the potential worsening of complications in patients with severe hyperglycemia. Therefore, this study aimed to examine whether chronic low‐intensity exercise training may prevent severe hyperglycemia‐induced capillary regression of skeletal muscle in non‐obese type 2 diabetes. Non‐diabetic Sprague Dawley rats were assigned to a control (Con) group and an exercise (Ex) group. Likewise, spontaneously diabetic Torii rats were assigned to a diabetic sedentary (DM) group or a diabetic exercise (DMEx) group. Rats in the Ex and DMEx groups were placed on a motor‐driven treadmill running at low speed (15 m/min) for 60 min/day, 5 days/week, for 14 weeks. Serum glucose levels were significantly increased in the DM group, but not in the DMEx group. Although the capillary‐to‐fiber ratio in the plantaris muscle was significantly lower in the DM group compared to the control group, the ratio in the DMEx group was significantly higher compared to the DM group. Moreover, the succinate dehydrogenase activity and expression levels of vascular endothelial growth factor and peroxisome proliferator‐activated receptor γ coactivator‐1α (PGC‐1α) were reduced in the plantaris muscle of the DM group. However, those in the DMEx group were significantly higher than those in the DM group. These results indicate that low‐intensity chronic endurance exercise training has the potential to prevent the progression of capillary regression in the skeletal muscles of non‐obese type 2 diabetes patients with severe hyperglycemia.

## Graphical abstract

Capillary regression may lead to a decrease in vascular endothelial growth factor expression due to severe hyperglycemia in the present study. Low‐intensity endurance exercise training prevented capillary regression and decreased mitochondrial oxidative capacity in the skeletal muscle of rats with non‐obese type 2 diabetes‐related severe hyperglycemia. Low‐intensity endurance exercise may have the potential to become an effective therapeutic intervention for the prevention of diabetes‐related complications in severe hyperglycemia patients for whom high‐intensity exercise is contraindicated.

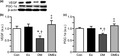

## 1 INTRODUCTION

Diabetes mellitus (DM) is a widely prevalent metabolic disorder that is associated with a marked increase in hyperglycemia‐induced diabetic complications (Tanaka et al., 2019; Zhao et al., 2014). Hyperglycemia leads to diverse complications such as muscle atrophy (Tanaka et al., 2019), insulin resistance, and a decline in the number of capillaries in skeletal muscle (Lillioja et al., 1987). In particular, type 2 diabetes is, in essence, a vascular disease that is frequently associated with capillary regression, which is a diabetic complication (Ko et al., 2010). Capillary regression of skeletal muscle is often evaluated as a decrease in capillary number (Gute et al., 1994; Koves et al., 2005). The capillary number of skeletal muscle plays an important role in supplying oxygen and nutrients to, and removing waste products from, muscle cells (Padilla et al., 2006). Therefore, it has been suggested that it is important to prevent hyperglycemia and hyperglycemia‐induced capillary regression for diverse complications in skeletal muscle.

A previous study suggested that the decrease in vascular endothelial growth factor (VEGF) expression is one of the key factors in capillary regression (Tang et al., 2004). VEGF is well‐known as a major angiogenic growth factor (Ferrara, 2001). In addition, previous studies have suggested that VEGF expression is decreased in diabetes (Kivela et al., 2006; Rivard et al., 1999). Therefore, the prevention of diabetes‐related decreased VEGF expression may prevent capillary regression. Hyperglycemia is also evoked by a reduction in the expression level of the transcriptional coactivator, peroxisome proliferator‐activated receptor‐gamma coactivator‐1α (PGC‐1α) (Mootha et al., 2003; Patti et al., 2003). Recently, PGC‐1α has attracted attention as a key player in the regulation of mitochondrial biogenesis (Arany et al., 2008) and mitochondrial oxidative capacity (Mootha et al., 2004; Pagel‐Langenickel et al., 2008). However, hyperglycemia causes decreased mitochondrial oxidative capacity through decreased PGC‐1α expression (Nagatomo et al., 2011; Nakamoto & Ishihara, 2020). In addition, PGC‐1α strongly correlates with VEGF expression and angiogenesis in skeletal muscles (Arany et al., 2008). Therefore, the inhibition of hyperglycemia‐induced decreased PGC‐1α expression may prevent the development of hyperglycemia‐induced capillary regression in diabetes via improved mitochondrial oxidative capacity.

Endurance exercise training, which is known to prevent the development of type 2 diabetes in humans (Knowler et al., 2002) and rodents (Pold et al., 2005), is recommended for the prevention of type 2 diabetes and/or its complications, such as preventing capillary regression in the skeletal muscle (Kondo et al., 2015). In general, endurance exercise training increases the capillary number of skeletal muscle via increased VEGF expression (Hermansen & Wachtlova, 1971; Jensen et al., 2004; Poole & Mathieu‐Costello, 1996; Richardson et al., 1999), thereby preventing diabetes‐related capillary regression.

Although endurance exercise is effective for diabetes‐related capillary regression, it is difficult to prescribe high‐intensity endurance exercise for diabetic patients due to the potential worsening of complications in patients with severe hyperglycemia (American Diabetes, 2016). Therefore, endurance exercise must be set to low intensity to prevent capillary regression in patients with severe non‐obese type 2 diabetes. Our previous study suggested that low‐intensity endurance exercise prevents capillary regression in non‐severe hyperglycemia‐related diabetes rat models (Goto‐Kakizaki rat) (Kondo et al., 2015). However, the previous study did not use severe hyperglycemia‐induced rat models and did not demonstrate the effects of low‐intensity exercise on severe hyperglycemia. We hypothesized that low‐intensity endurance exercise might prevent severe hyperglycemia‐induced capillary regression via inhibition of mitochondrial oxidative capacity in non‐obese type 2 diabetes as well as non‐severe hyperglycemia diabetes. If this study is validated, new therapeutic interventions for non‐obese patients with severe diabetes can be established. Therefore, the purpose of the present study was to examine whether chronic low‐intensity exercise training may prevent severe hyperglycemia‐induced capillary regression of skeletal muscle in a non‐obese type 2 diabetic rat model and whether factors related to the inhibition of severe hyperglycemia through the improvement of mitochondrial oxidative capacity might be involved in the preventive effects.

## 2 MATERIALS AND METHODS

### 2.1 Experimental design

Eleven‐week‐old male Sprague Dawley (SD) rats were purchased from CLEA Japan, Inc. and assigned to either a non‐diabetes control (Con) group or a non‐diabetes exercise (Ex) group. Spontaneously Diabetic Torii (SDT) rats were assigned to either a diabetes mellitus (DM) group or a DM exercise (DMEx) group (*n* = 5 in each group). SDT rats have been established as a model for non‐obese and severe hyperglycemia‐induced type 2 diabetes (Shinohara et al., 2000). The rats in the Ex and DMEx groups were running on a motor‐driven treadmill at low speed (15 m/min) for 60 min/day, 5 days/week, for 14 weeks after being familiarized with the treadmill for 5–10 min per day for a period of 1 week. The levels of blood lactate in the Ex and DMEx groups were measured from the tail vein using a blood lactate test meter (Lactate Pro; Arkray, Shiga, Japan) before and after exercise. The exercise was considered as aerobic in terms of intensity because the levels of blood lactate did not change significantly (1.30 ± 0.21 mmol/L <2 mmol/L) before and after exercise. The animals had access to food and water ad libitum. All rats were maintained at 22 ± 2°C with a light–dark cycle of 12 h. All experiments were conducted in accordance with the National Institutes of Health (NIH) Guide for the Care and Use of Laboratory Animals (National Research Council, 1996) and approved by the Animal Care and Use Committee of Kobe University, Japan.

### 2.2 Biochemical analyses of blood

Every 2 weeks from 10 weeks to 24 weeks of age, glucose levels were measured using a blood glucose monitoring system (Precision Xceed, Abbot Laboratories, Illinois, USA) from blood samples obtained from the tail vein in the non‐fasting state.

At 25 weeks of age, after all the rats were anesthetized with pentobarbital sodium (50 mg/kg, *i*.*p*.), blood samples were obtained from the abdominal veins after 9 h of fasting. The animals were then euthanized by an overdose of sodium pentobarbital. Hemoglobin (Hb)A1c was then measured using a DCA Vantage Analyzer (Siemens Medical Solutions Diagnostics). Serum was obtained by centrifugation (3000 × *g* for 10 min) at room temperature and stored at −80°C until further analyses. Serum glucose was measured using the Glucose CII‐Test Wako kit (Wako Pure Chemical Industries).

### 2.3 Histological analyses

Twenty‐four hours after the final exercise session and after blood sampling, the plantaris muscle was excised, quick‐frozen in isopentane, pre‐cooled in liquid nitrogen, and stored at −80°C. Thereafter, the muscle was sliced into 12‐μm‐thick transverse sections using a cryostat microtome (CM3050; Leica Microsystems) at −20°C, and the sections were dried at room temperature for 30 min. Several sections were stained to determine the levels of alkaline phosphatase (AP) to visualize capillaries in the skeletal muscle. For AP staining, the sections were incubated for 60 min at 37°C in 0.1% 5‐bromo‐4‐chloro‐3‐indolyl phosphate *p*‐toluidine salt and 0.1% nitro blue tetrazolium in 0.2 M borate buffer. The sections were observed under a light microscope (×400: BX51; Olympus, Tokyo, Japan), and images were obtained with a CCD camera (VB‐7000; Keyence, Osaka, Japan). The capillary‐to‐fiber ratio (C/F) ratio was measured in microscopic images selected at random from AP stained sections. The C/F ratio in the deep layer of the plantaris muscle (two images in each rat) was determined by counting all the capillaries and fibers in a microscopic image. All measurements were subsequently calculated using the ImageJ software program (NIH).

The level of succinate dehydrogenase (SDH) activity, an indicator of mitochondrial oxidative capacity (Nakatani et al., 1999; Wust et al., 2009), was measured in the stained sections. For SDH staining, the sections were incubated for 45 min at 37°C in 0.05% nitroblue tetrazolium and 0.05 M sodium succinate in 0.05 M phosphate buffer (pH 7.5). Cross‐sectional images of tissues were visualized with a light microscope, and images were obtained with a CCD camera. Microscopic images were randomly selected from each section, and all muscle fibers in each image were analyzed to determine SDH activity in the plantaris muscle. SDH activity was calculated as the mean optical density using the Image J software program (NIH). All values are shown as fold changes relative to the Con group.

### 2.4 Western blot analyses

The frozen muscle samples were homogenized in ice‐cold homogenizing buffer (50 mM Tris–HCl, pH 7.8) containing a protease inhibitor cocktail (1:200, P8340; Sigma Chemicals). Following this, the homogenate was centrifuged at 15,000 *g* for 15 min at 4°C, and the total protein concentration was determined using a protein determination kit (Bio‐Rad Laboratories). The homogenates were solubilized in sample loading buffer (62.5 mM Tris–HCl, pH 6.8, 2% sodium dodecyl sulfate (SDS), 10% glycerol, 5% 2‐mercaptoethanol, and 0.02% bromophenol blue), and were boiled for 10 min at 80°C. Equal amounts of proteins were separated by SDS‐polyacrylamide gel electrophoresis and then transferred to a polyvinylidene difluoride membrane. Following an overnight blocking step in 5% skim milk in phosphate‐buffered saline with Tween 20 (PBST), the membranes were incubated with anti‐VEGF antibody (1:1000 in PBST, sc‐7269; Santa Cruz Biotechnology) and anti‐PGC‐1α antibody (1:1000 in PBST, sc‐13067, Santa Cruz Biotechnology) at 4°C. Following overnight incubation, the membranes were incubated for 60 min at room temperature with anti‐mouse or anti‐rabbit secondary antibodies (1:10,000 in PBST). The signals were detected using the enhanced chemiluminescent Prime Western Blotting Detection System (GE Healthcare) and analyzed with an image reader (LAS‐1000, Fujifilm). β‐actin was used as an internal control.

### 2.5 Statistical analyses

All data are represented as the mean ± standard error of the mean. Two‐way analysis of variance (ANOVA) was used to analyze the blood glucose levels among the Con, Ex, DM, and DMEx groups followed by Tukey's post hoc tests to determine specific group differences. One‐way ANOVA was used to analyze the capillary‐to‐fiber ratio, SDH activity, and western blot protein expression levels among the Con, Ex, DM, and DMEx groups, followed by Tukey's post hoc tests to determine specific group differences. Statistical significance was set at *p* < 0.05.

## 3 RESULTS

### 3.1 Blood glucose level

**FIGURE 1:**
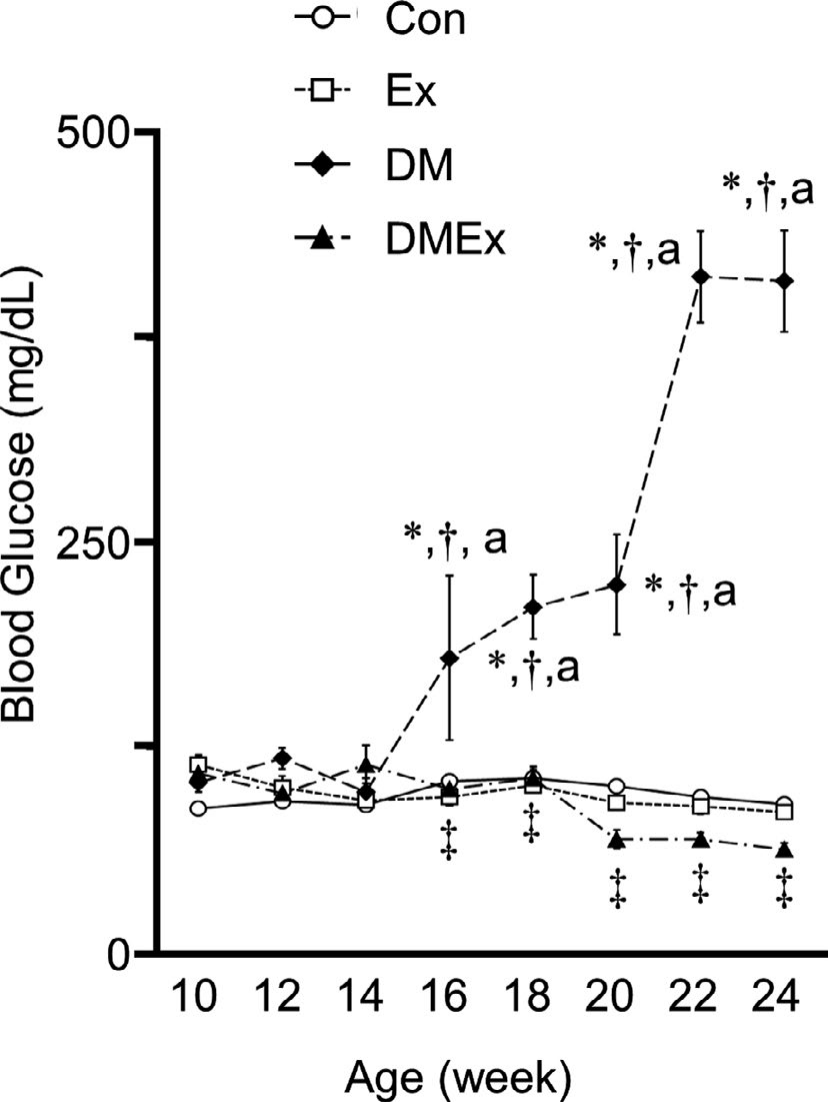
Blood glucose level. The values of blood glucose are presented as the mean ± standard error of the mean (SEM). ○, control group; □, exercise group; ◆, diabetes group; ▲, diabetes with the exercise group. *, †, and ‡ indicate significant differences in the Con, Ex, and DM groups, respectively, at *p* < 0.05. a is significantly different from the DM group, 14 weeks of age at the time point of the same group at *p* < 0.05. Con, control group; DM, diabetes group; Ex, exercise group

Blood glucose levels are shown in Figure 1. There was no significant difference in blood glucose levels between the DM and Con groups until 14 weeks of age. However, the blood glucose level in the DM group increased markedly from 16 to 22 weeks of age. Blood glucose levels were significantly higher in the DM group than in the Con group at 16 to 24 weeks of age. In contrast, the blood glucose levels in the DMEx group were significantly lower than those in the DM group at 16 to 24 weeks of age. Furthermore, there were no significant differences between the Con, Ex, and DMEx groups during the experimental period.

### 3.2 Body weight, relative muscle mass to body weight, serum glucose, and HbA1c

Body weight, the muscle‐to‐body weight ratio of each muscle, serum glucose, and HbA1c are shown in Table 1. There was no significant difference in body weight between the groups. The muscle mass to body weight ratio in the DM group was significantly lower than that in the Ex group. Although serum glucose and HbA1c levels were significantly higher in the DM group than in the Con and Ex groups, they were significantly lower in the DMEx than in the DM group at 25 weeks of age. Moreover, there were no significant differences between the Con, Ex, and DMEx groups.

**TABLE 1:** Body weight (g), relative muscle mass (mg/g), serum glucose (mg/dL), and hemoglobin A1c (%) at the end of the exercise

	Body weight (g)	Relative muscle mass to body weight (mg/g)	Serum glucose (mg/dL)	HbA1c (%)
Con	471 ± 9	1.07 ± 0.04	217.4 ± 18.5	3.1 ± 0.1
Ex	452 ± 7	1.11 ± 0.03	186.1 ± 15.0	3.0 ± 0.1
DM	436 ± 13	0.89 ± 0.06^†^	443.8 ± 20.1*^,†^	7.0 ± 0.3*^,†^
DMEx	469 ± 11	1.07 ± 0.01	231.9 ± 24.5^‡^	3.2 ± 0.1^‡^

The values of body weight, relative muscle mass, serum glucose, and hemoglobin A1c are presented as the mean ±standard error of the mean (SEM).

Abbreviations: Con, control group; Ex, exercise group; DM, diabetes group; DMEx, diabetes with the exercise group.

*, †, and ‡ indicate significant differences in the Con, Ex, and DM groups, respectively, at *p* < 0.05.

### 3.3 Capillary‐to‐fiber ratio

**FIGURE 2:**
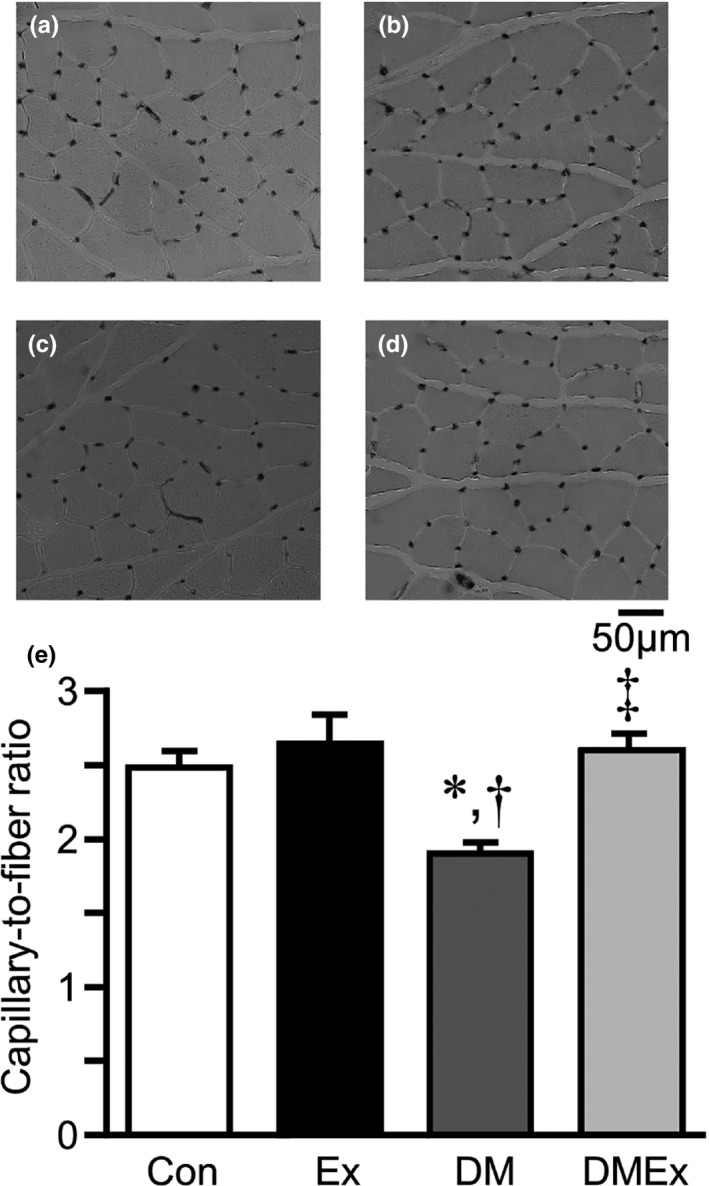
Capillary to muscle fiber ratio in plantaris muscle. Alkaline phosphatase staining of the plantaris muscle (a–d). (a) Control group; (b) Exercise group; (c) Diabetes group; (d) Diabetes with the exercise group. The capillaries are arranged around the muscle fibers and visible as dark spots. The number of capillaries to muscle fiber ratio (C/F ratio) in the plantaris muscle (e). The values of the C/F ratio are presented as mean ±standard error of the mean (SEM). *, †, and ‡ indicate significant differences from the Con, Ex, and DM groups, respectively, at *p* < 0.05. Con, control group; DM, diabetes group; Ex, exercise group . Scale bar = 50 μm

Representative AP staining images in the plantaris muscle of the Con, Ex, DM, and DMEx groups are shown in Figure 2a‐d. Capillaries were arranged around the muscle fibers and visualized as dark spots. The C/F ratio in the DM group was significantly lower than that in the Con and Ex groups (Figure 2e). However, the C/F ratio in the DMEx group was significantly higher than that in the DM group.

### 3.4 SDH activity

**FIGURE 3:**
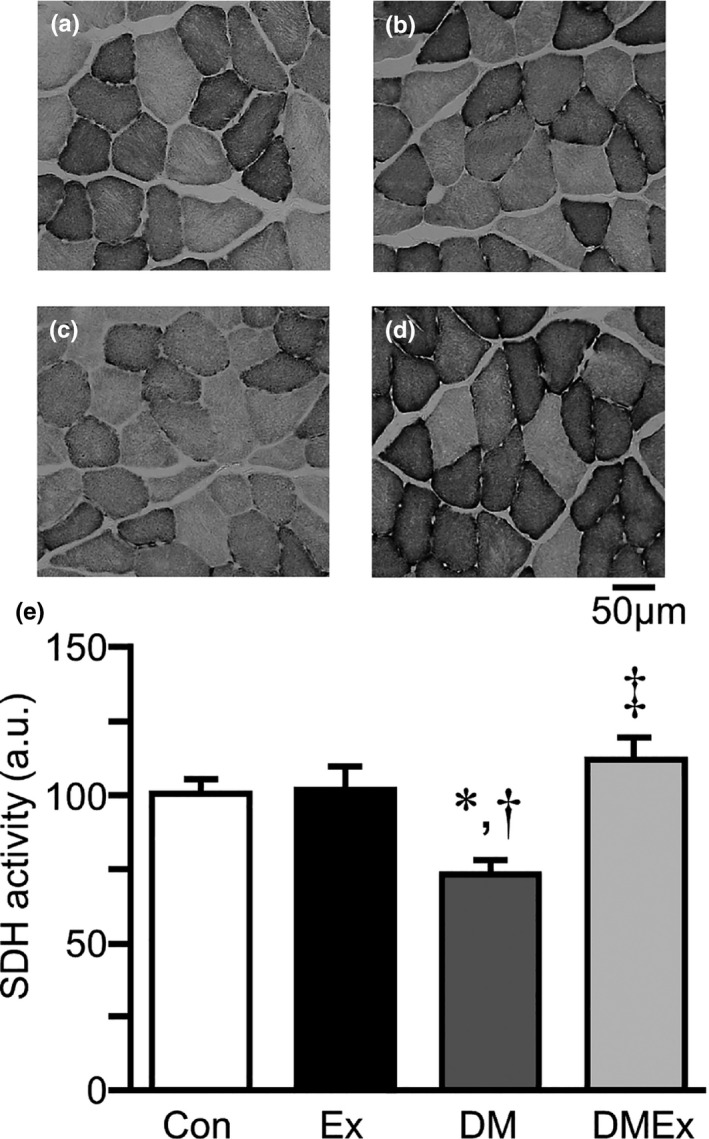
Succinate dehydrogenase (SDH) activity of plantaris muscle. SDH staining of transverse sections of the plantaris muscle (a–d). (a) Control group; (b) Exercise group; (c) Diabetes group; (d) Diabetes with exercise group. Staining intensity is directly related to SDH activity; darker muscle fibers indicate higher activity. SDH activity in the plantaris muscle (e) The values of SDH activity are presented as the mean ± standard error of the mean (SEM). All values indicate fold changes relative to the control group. a.u., arbitrary unit. *, †, and ‡ indicate significant differences from the Con, Ex, and DM groups, respectively, at *p* < 0.05. Con, control group; DM, diabetes group; Ex, exercise group. Scale bar = 50 μm

Representative SDH staining images in the plantaris muscle of the Con, Ex, DM, and DMEx groups are shown in Figure 3a‐d. The SDH activity of muscle fibers in the DM group was significantly lower than that in the Con group (Figure 3e). In contrast, SDH activity was significantly higher in the DMEx group than in the DM group.

### 3.5 VEGF and PGC‐1α expression levels in plantaris muscles

**FIGURE 4:**
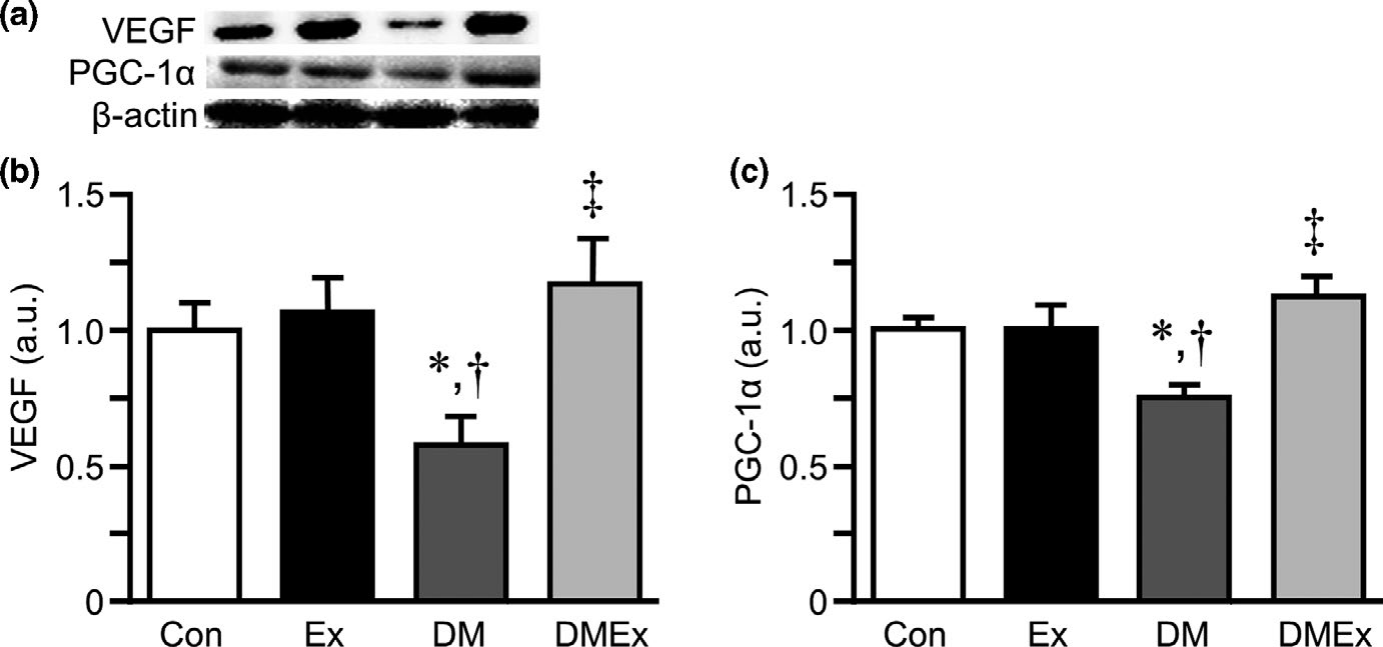
The expression of vascular endothelial growth factor (VEGF) and peroxisome proliferator‐activated receptor γ coactivator‐1α (PGC‐1α) in the plantaris muscle. Representative western blot images of VEGF and PGC‐1α in the plantaris muscle (a). Representative western blots indicate the expression of VEGF (b) and PGC‐1α (c) proteins in the plantaris muscle. The values of the expression levels are presented as the mean ± standard error of the mean (SEM). a.u., arbitrary unit. *, †, and ‡ indicate significant differences from the Con, Ex, and DM groups, respectively, at *p* < 0.05. Con, control group; DM, diabetes group; Ex, exercise group

Representative western blot images of VEGF and PGC‐1α in the plantaris muscle are shown in Figure 4c. The expression level of VEGF protein was significantly lower in the DM group than in the Con group. In contrast, the expression level of VEGF protein was significantly higher in the DMEx group than in the DM group (Figure 4a). The expression level of PGC‐1α protein in the DM group was significantly lower than that in the Con group, whereas it was higher in the DMEx group than in the DM group (Figure 4b).

## 4 DISCUSSION

In the present study, we found that low‐intensity endurance exercise in non‐obese DM prevented capillary regression and maintained the expression level of VEGF in the plantaris muscle of the control group, which contributed to the prevention of hyperglycemia‐induced capillary regression. In addition, endurance exercise inhibited the decrease in the mitochondrial oxidative capacity that is typically associated with DM, which contributed to the inhibition of severe hyperglycemia. These results suggest that low‐intensity endurance exercise training can both alleviate capillary regression and decrease mitochondrial oxidative capacity via inhibition of severe hyperglycemia in the skeletal muscles of rats with non‐obese type 2 diabetes‐related severe hyperglycemia.

It was observed that the diabetes group exhibited severe hyperglycemia and capillary regression as well as decreased expression levels of VEGF in the plantaris muscle. In several studies, the capillary number of skeletal muscle was reduced in diabetes (Kivela et al., 2006; Marin et al., 1994; Mathieu‐Costello et al., 2003; Sexton et al., 1994), which is expected since VEGF expression levels regulate capillary number (Liu et al., 2018). In addition, previous studies have suggested that VEGF expression levels are decreased in diabetic conditions as was observed in the present study (Hazarika et al., 2007; Liu et al., 2018). In contrast, our previous study suggested that non‐severe hyperglycemia diabetes model rats did not show decreased VEGF expression (Kondo et al., 2015). VEGF expression depends on capillary regression through endothelial cell disruption (Tsurumi et al., 1997). In addition, hyperglycemia induces a decrease in VEGF expression through endothelial cell disruption (Brownlee, 2001; Yang et al., 2008). Furthermore, our previous study using non‐severe hyperglycemia diabetes rat models also showed that the capillary number did not decrease, as well as not show a decrease in VEGF expression (Kondo et al., 2015). Decreased capillary number was related to endothelial cell disruption (Fujino et al., 2005). Therefore, these results suggest that capillary regression may lead to a decrease in VEGF expression due to severe hyperglycemia in the present study, consistent with a previous study using obese diabetes conditions (Hazarika et al., 2007; Liu et al., 2018). In addition, the decreased capillary number reported here, which is different from our previous study, may be related to a severe hyperglycemia‐induced decrease in VEGF expression.

Conversely, low‐intensity endurance exercise training prevented capillary regression in the plantaris muscle in non‐obese diabetes‐related severe hyperglycemia. In addition, exercise training in diabetes prevented decreased mitochondrial oxidative capacity. Moreover, exercise training inhibited hyperglycemia in non‐obese patients with diabetes (Kishimoto et al., 2002). These results suggest that the prevention of capillary regression may be inhibited in cases of non‐obese diabetes‐related hyperglycemia via exercise. Muscle contraction during endurance exercise was shown to increase glucose uptake during exercise and decrease blood glucose levels via increased glucose consumption in the skeletal muscle, even in low‐intensity exercise (Michishita et al., 2008; Mul et al., 2015). In the present study, a regular exercise regime was set and every exercise‐induced decrease in blood glucose level was temporary. Therefore, the exercise strategy prevented severe hyperglycemia‐induced capillary regression via a muscle contraction‐induced decrease in blood glucose level on a regular basis in hyperglycemic rats.

Numerous studies have shown that exercise training strongly induces an increase in PGC‐1α expression in skeletal muscle (Baar et al., 2002; Koves et al., 2005; Norrbom et al., 2004). PGC‐1α expression was maintained in diabetes even with low‐intensity exercise (Kondo et al., 2015). The inhibition of decreased PGC‐1α expression caused an improvement in decreased mitochondrial oxidative capacity in the skeletal muscle via inhibition of increased blood glucose levels in diabetes conditions (Nakamoto & Ishihara, 2020). In addition, glucose demand for skeletal muscle is related to mitochondrial oxidative capacity (Booth et al., 2015). Therefore, the prevention of decreased mitochondrial oxidative capacity could be caused by the inhibition of decreased PGC‐1α expression via exercise effects. Furthermore, capillaries around the skeletal muscle have a role in supplying glucose to the skeletal muscle (Padilla et al., 2006). Hence, in the present study, the low‐intensity exercise could lead to the inhibition of severe hyperglycemia via both the prevention of capillary regression‐maintained glucose supply and the prevention of decreased mitochondrial oxidative capacity‐related maintained glucose consumption. In addition, the inhibition of hyperglycemia via the prevention of decreased mitochondrial oxidative capacity may induce further prevention of hyperglycemia‐induced capillary regression.

This study has several limitations. First, the current findings showed that low‐intensity exercise can prevent capillary regression due to non‐obese type 2 diabetes‐related severe hyperglycemia; however, a comparison between low‐ and high‐intensity exercise was not performed. Second, in this study we focused on a fast‐twitch muscle (plantaris), not on a slow‐twitch muscle (e.g., soleus muscle); however, a comparison between fast‐ and slow‐twitch muscle was not analyzed. This study focused on fast‐twitch muscle because a percentage of the fast muscle fiber increases in the diabetic muscle through altered fiber distribution (Yasuda et al., 2002). Fast muscle fiber results in increased oxidative stress through the accumulation of advanced glycation end products (AGEs) due to a low tolerance to oxidative stress (Hagiwara et al., 2009; Snow & Thompson, 2009; Tanaka et al., 2020). Increased oxidative stress induces capillary regression (Hirayama et al., 2017). Therefore, the fast‐twitch muscle causes capillary regression to a greater extent compared with the slow‐twitch muscle. In addition, the fast‐twitch muscle results from harmful effects such as muscle atrophy and dysfunction of mitochondrial metabolism due to a low tolerance to hyperglycemia induced‐oxidative stress compared with slow‐twitch muscle in diabetic conditions (Ciciliot et al., 2013; Nakamoto & Ishihara, 2020; Tanaka et al., 2019). Furthermore, fast‐twitch muscle in the hindlimb plays an important role as locomotor and for physical activity (Ikezoe et al., 2011). Therefore, it would be especially important to prevent harmful effects in the fast‐twitch muscle in the hindlimb. In addition, this study was unable to clarify the changes in the properties of muscle type. Previous studies suggested that oxidative capacity was dependent on fiber type properties (Nakamoto & Ishihara, 2020; Qatamish et al., 2020). The decrease in oxidative capacity caused an increase in fast‐glycolytic muscle fibers (type IIB) and decreased fast‐oxidative muscle fibers (type IIA) in diabetic rats (Nakamoto & Ishihara, 2020). In addition, exercise inhibited the increase in type IIB fibers (Nakamoto & Ishihara, 2020). Therefore, the preventive effects of decreased oxidative capacity may result in muscle fiber properties inhibiting the increase of fast‐glycolytic muscle fibers in the present study. Third, the present study focused on the mechanism of how low‐intensity exercise regulates capillary regression and decreased mitochondrial oxidative capacity in the skeletal muscle of rats with non‐obese type 2 diabetes‐related severe hyperglycemia. However, we could not clarify the complete mechanism in the present study. Hence, further studies will be required to clarify these issues.

## 5 CONCLUSION

Low‐intensity endurance exercise training prevented capillary regression and decreased mitochondrial oxidative capacity in the skeletal muscle of rats with non‐obese type 2 diabetes‐related severe hyperglycemia. These results suggest that low‐intensity endurance exercise may have the potential to become an effective therapeutic intervention for the prevention of diabetes‐related complications in severe hyperglycemia patients for whom high‐intensity exercise is contraindicated.

## CONFLICT OF INTEREST

No potential conflicts of interest were reported by the authors.
